# Basic Concepts in Metastatic Cardiac Disease

**DOI:** 10.4021/cr155w

**Published:** 2012-03-20

**Authors:** Panagiotis J. Vlachostergios, Danai D. Daliani, Christos N. Papandreou

**Affiliations:** a Department of Medical Oncology, University Hospital of Larissa, University of Thessaly School of Medicine, Biopolis 41110, Larissa, Greece

**Keywords:** Heart metastasis, Cancer

## Abstract

The involvement of the heart in metastatic cancer is a rare clinical diagnosis, as it may be asymptomatic or symptoms, when present, may be attributed to other causes. Issues regarding incidence, intracardiac location, clinical presentation, diagnosis and treatment of metastatic cardiac tumors will be discussed here.

## Introduction

Metastatic tumours of the heart although being rare, present in a much greater incidence compared to primary tumors (at a ratio of at least 30 to 1) and originate from any neoplastic tissue with the potential to metastasise. The most common, among the latter, are lung, breast and oesophageal cancers, lymphomas, leukemias and finally melanoma, which has the highest rate of cardiac metastasis [1]. Based on data from postmortem studies of Bussani et al. during the period from 1994 to 2003, the incidence of cardiac metastases involves 9.1% of early-stage cancers and 14.2% of metastatic. The most common sites of origin include the lung (pleural mesothelioma 48.4%, adenocarcinoma 21%, squamous-cell carcinoma 18.2%), skin (melanoma 27.8%), breast (15.5%) and undifferentiated carcinomas (19.5%) [2].

## Intracardiac Location

By location, approximately two thirds of cardiac metastases involve the pericardium, and one third resides in the myocardium or/and the epicardium, with only 5% of cases involving the endocardium. This distribution is determined by the different ways in which cancer is spreading [3]. Pericardial infiltration is either a result of local direct invasion by intrathoracic tumors or derives from myocardial involvement and furthermore through retrograde lymphatic spreading from mesothoracic and tracheal nodes. Myocardial or epicardial metastasis is usually hematogenous or lymphogenous whereas the endocardium is generally invaded through systematic circulation and in fewer cases through myocardial involvement [2, 3].

## Clinical Presentation

The clinical manifestations vary according to the intracardiac location of metastasis, but in general they are rarely observed, only at 10%, particularly in patients with advanced disease [1]. Pericardial involvement may cause dyspnea, hypotension, tachycardia and even signs of cardiac tamponade due to pericardial effusion. Myocardial infiltration is associated with arrythmias and if generalized it may be responsible for congestive heart failure and systolic or diastolic dysfunction. In rare cases, a neoplastic thrombus inside the coronary circulation may cause an acute myocardial infarction through invasion, or strangulation of coronary arteries by a massive pericardial effusion. In cases of intraventricular location of the neoplastic mass, an obstruction is expected to damage the tricuspid or mitral valve function (with a clinical presentation resembling to that of tamponade) or spread neoplastic emboli to the arterial circulation of the lungs after being ruptured. Moreover, the organization of neoplastic thrombi inside the right atrium or ventricule may facilitate the dispersal of cancer cells to distant metastatic sites [2].

The right-sided cardiac metastases are more common than the left-sided, as warranted by lower right flow and systolic pressure that favour the anchorage of neoplastic cells. Conversely, invasion to the heart valves is rare due to constituent movement and lack of vascularization [2].

## Diagnosis

The diagnosis of cardiac metastases is not usually guided by specific signs or laboratory findings. Intraventricular invasion may be responsible for systolic or/and diastolic murmurs of stable or ranging intensity, gallop rhythm, congestive heart failure, pericardial friction sound and attenuated cardiac sounds in case of pericardial effusion [3]. Electrocardiograms may reveal conduction disorders, atrial or ventricular arrythmias or ST-segment abnormalities due to pericardial effusion. Enlarged cardiac silouette and pleural effusion are common findings in chest x-rays. The gold standard for diagnosis is two-dimentional echocardiography, often showing pericardial thickening or fluid, mobility abnormalities of myocardial wall and intraventricular masses [3]. Transoesophageal ultrasound is superior to transthoracic in diagnosing peri and para-cardial lesions. The roles of CT and MRI are complementary, contributing particularly to determination of exact location and composition of lesions [4]. Finally, the expanding use of positron emission tomography (PET) scan, seconded by CT, is expected to further increase the number of diagnosed cases [5].

## Differential Diagnosis

The differential diagnosis of an intra-cardiac mass includes the existence of thrombus, vegetation or non-neoplastic conditions, such as pericardial cyst, teratoma, lipomatous hypertrophy of the atrial septum, papillary fibroelastomas and sarcoid granuloma [1]. It is noteworthy that myocardial and pericardial metastases must always be taken into consideration when differential diagnosing patients with acute myocardial infarction and pericardial effusion, respectively. Moreover, the contribution of iatrogenic cardiac damage is not negligible, either as radiotherapy-induced pericardial effusion, compressive pericarditis and myocardial or valve fibrosis or as chemotherapy (antracycline) - induced cardiomyopathy. Thrombocytopenia, neutropenia and corticosteroid therapy may also lead to potentially threatening conditions such as cardiac hemorrhage, abscess and adipose infiltration [1].

## Treatment Options

Given the advanced stage of disease at the time of cardiac metastases, the management of patients is normally palliative. Surgical excision is only indicated for solitary lesions causing obstruction of intra-ventricular or valvular flow in patients of good prognosis with total resection of the primary tumour [2]. Even in such cases, total excision is often infeasible, with high post-surgical morbidity. Coil embolization of the supplying coronary artery branch is an alternative choice in circumscribed lesions. Malignant pericardial effusion is usually manageable with local irradiation [6] or systemic chemotherapy, otherwise the patient may be subjected to local infusion of chemotherapeutic agents (such as bleomycin, thiotepa, cis-platin, mitoxantrone, mitomycin C, 5-FU) or radioisotopes (^32^P) and in rare cases drainage through subxiphoid pericardiotomy or pericardial window [1, 2].

It seems that the incidence of cardiac metastases is rising [6], principally due to the application of new, more efficacious chemotherapeutic agents and irradiation techniques, both contributing to elongation of overall survival and consequently maximization of the possibility for presentation of metastatic disease. Therefore, oncologists and treating physicians should be vigilant for symptoms and signs compatible with cardiac infiltration, given the poor prognosis of such patients.
